# A Novel Trivalent BVDV mRNA Vaccine Displayed by Virus-like Particles Eliciting Potent and Broad-Spectrum Antibody Responses

**DOI:** 10.3390/vaccines13070691

**Published:** 2025-06-26

**Authors:** Shi Xu, Jing Li, Mengwei Xu, Yafei Cai, Yingjuan Qian, Rui Liu, Qing He, Caiyi Fei, Aili Wang, Keyue Ruan, Shang Liu, Wei Geng, Xu Gao, Huiling Chen, Tiyun Han

**Affiliations:** 1 Nanjing Chengshi (TheraRNA) Biomedical Technology Co., Ltd., Nanjing 210000, China; xushi@therarna.cn (S.X.); li.jing@therarna.cn (J.L.); xu.mengwei@therarna.cn (M.X.); liurui@therarna.cn (R.L.); he.qing@therarna.cn (Q.H.); feicaiyi@therarna.cn (C.F.); wang.aili@therarna.cn (A.W.); ruan.keyue@therarna.cn (K.R.); liu.shang@therarna.cn (S.L.); geng.wei@therarna.cn (W.G.); 2 College of Animal Science and Technology, Nanjing Agricultural University, Nanjing 210095, China; ycai@njau.edu.cn (Y.C.); 15122425@stu.njau.edu.cn (X.G.); 3 MOE Joint International Research Laboratory of Animal Health and Food Safety, College of Veterinary Medicine, Nanjing Agricultural University, Nanjing 210095, China; yqian@njau.edu.cn; 4 Department of Hematology, Lanzhou University Second Hospital, Lanzhou 730030, China

**Keywords:** bovine viral diarrhea virus, mRNA vaccine, envelope glycoprotein E2, artificial virus-like particle, antibody responses

## Abstract

**Background/Objectives**: Bovine viral diarrhea virus (BVDV) causes significant economic losses in the cattle industry worldwide. The current vaccines have limited efficacy against diverse BVDV genotypes. Currently, multi-antigen target design and nanocarrier display technologies can provide ideas for broad-spectrum and efficient BVDV vaccine design. **Methods**: Here we developed a trivalent mRNA vaccine encoding the domains I-II of envelope glycoprotein E2 from three BVDV genotypes (3E2), introduced with bovine IgG1 Fc (bFc), STABILON (hStab), and artificial virus-like particle (ARVLP) containing CD80 transmembrane (TM) domain, FcγRII cytoplasmic domain, and WW domain of ITCH. Then, in vitro expression, in vivo immunogenicity and neutralizing antibody analysis were performed to evaluate the vaccines. **Results**: The in vitro expression results showed that bFc and hStab dramatically enhanced antigen expression and immunogenicity. In addition, the ARVLP further enhanced the secretion and potency of neutralizing antibodies. Finally, the immunogenicity of the bFc_BVDV_3E2_ARVLP_hStab mRNA vaccine was evaluated in mice, guinea pigs, and lactating goats and high levels of neutralizing antibodies against all three BVDV genotypes were detected. **Conclusions**: Our trivalent design strategy with bFc, hStab, and ARVLP shows highly efficient expression as well as strong immunogenicity and provides a promising approach for next-generation BVDV vaccines with broader and stronger protection.

## 1. Introduction

Bovine viral diarrhea virus (BVDV) is a major pathogen affecting cattle worldwide, causing significant economic losses estimated at USD 400–750 per animal through reduced milk production, reproductive failure, and increased susceptibility to secondary infections [1,2]. BVDV exists as two major genotypes (BVDV-1 and BVDV-2), with BVDV-1 further subdivided into several subtypes including BVDV-1a and BVDV-1b [3]. In China, BVDV-1a, BVDV-1b, and BVDV-2 are the predominant circulating strains [4]. Current commercial vaccines, including inactivated and modified live vaccines, have several critical limitations: they typically provide limited cross-protection against different BVDV genotypes, require multiple doses to achieve protective immunity, and may have safety concerns regarding the incomplete inactivation or potential reversion to virulence [5]. In addition, the manufacturing process for traditional vaccines is time-consuming and may not respond quickly to emerging variants.

The emergence of mRNA vaccine technology represents a promising platform to address these challenges. mRNA vaccines have two advantages over traditional vaccine approaches: they can be rapidly produced under AI-guided protein design, allowing precise control of antigen expression, and have demonstrated excellent safety profiles [6]. The success of mRNA vaccines against COVID-19 has validated this platform and accelerated its application to other pathogens [7]. Moreover, recent advances in mRNA modification, sequence optimization, and delivery systems have significantly improved the stability and immunogenicity of mRNA vaccines. For veterinary applications in particular, mRNA vaccines offer the potential for cost-effective production and simplified cold chain requirements [8]. However, the optimization of antigen design and delivery remains critical to achieving robust immune responses with mRNA vaccines, especially for complex viral antigens that require specific conformational epitopes for neutralization.

The BVDV envelope glycoprotein E2, as the major envelope protein, contains the primary neutralizing epitopes and is the primary research target for protective antibody responses against BVDV infection [9]. However, the structural basis for broad neutralization has remained elusive, hampering rational vaccine design efforts. Previous attempts to use full-length E2 or empirically selected fragments as vaccine antigens have yielded suboptimal results, likely due to improper protein folding or exposure of non-neutralizing epitopes [10]. Given the advances in protein structure prediction with AlphaFold 3, a structure-guided antigen design approach will provide a novel basis for the development of broadly protective vaccines [11].

To improve antigen expression and immunogenicity, we employed two innovative strategies based on recent advances in protein engineering and cell biology. We generated fusion constructs combining the E2 domains with the IgG1 Fc domain and stabilizing peptide sequences called STABILON [12,13]. The Fc fusion serves multiple purposes: it enhances protein secretion through improved folding and trafficking, increases half-life through FcRn-mediated recycling, and potentially enhances immune responses through Fc receptor engagement [12]. The addition of STABILON has been reported to increase the concentration of the corresponding mRNA and stabilize the short-lived fusion protein [13]. Meanwhile, the endocytosis prevention motif (EPM), low affinity immunoglobulin gamma Fc region receptor II (FcγRII) cytoplasmic domain, could prevent coated pit localization and endocytosis, and together with the ESCRT- and ALIX-binding region (EABR) could promote the secretion of self-assembling enveloped virus-like particles (eVLPs) expression [14]. The WW domain, also known as the RSP5 domain or the WWP repeating motif, is a modular protein domain that mediates specific interactions with protein ligands containing particular proline motifs [15]. The fused WW domain of the E3 ubiquitin protein ligase Nedd4 family proteins could be recognized by the late domain-containing protein Ndfip1, leading to ubiquitination and entry into exosomes [16]. In addition, the vectors, such as virus-like particle (VLP) or exosomes, could provide stable protection and antigen display to induce higher levels of humoral immune response. Therefore, we further introduced the artificial virus-like particle (ARVLP) elements containing CD80 transmembrane (TM) domain, FcγRII cytoplasmic tail, and WW domain of ITCH, the member protein of the Nedd4 family, into constructs to display antigens in the form of VLP to enhance their expression, secretion levels and immunogenicity.

Here we report the development and evaluation of a trivalent mRNA vaccine encoding three truncated E2 domains I-II of BVDV-1a, BVDV-1b, and BVDV-2 with stable IgG-like structures. Incorporating bFc, STABILON, and ARVLP elements into the vaccine construct significantly increased the secretion levels of the target antigen in vitro and the neutralizing antibody titers in vivo. After immunization of the optimized vaccine in mice and guinea pigs, sustained neutralizing antibody responses were found against BVDV, suggesting an effective display of the neutralizing epitopes in the truncated structure of the E2 domain I-II displayed by ARVLP. The presence of high levels of neutralizing antibodies against all three BVDV genotypes in guinea pigs and lactating goats indicated successful cross-protection induction. Finally, the vaccine was found to be comparably immunogenic and effective in different injection routes and when compared to the commercial vaccine in lactating goats.

## 2. Materials and Methods

### 2.1. Ethics Statement and Animals

The conducted animal experiments were approved by the Animal Ethics Committee of Lanzhou University Second Hospital, with the approval number D2023-398. Experimental procedures adhered strictly to the ARRIVE reporting guidelines and complied with national guidelines for the care and use of laboratory animals. Animals were housed under controlled conditions, with relative humidity maintained at 50–60% and a 12 h light/dark cycle. Food and water were provided with no restriction.

Sample size was decided in consideration with statistically significant and minimized animal use, especially large animals. A randomization sequence was generated using a computerized random number generator using the RAND function in Microsoft Excel. Animals were randomly assigned to each group in equal numbers, ensuring group sizes were balanced and comparable at baseline. To minimize potential confounders, we applied randomized treatment order, standardized measurement times, cage rotation, and blinded assessments; if not controlled, this should be stated explicitly. Animals were vaccinated on day 0 (prime) and day 14 (or 21) (boost), followed by serum collection on day 28 or 35 to assess antibody inhibition. These schedules are consistent with standard protocols for mRNA vaccines.

### 2.2. Vaccine Design and Molecular Engineering

To develop a trivalent mRNA vaccine targeting BVDV, we designed four different mRNA constructs encoding domains I-II of the E2 proteins from BVDV1a, BVDV1b, and BVDV2 strains. Each construct was engineered to enhance protein expression, stability, and immunogenicity through strategic molecular modifications. (1) The BVDV_3E2 construct: This construct consists of a fusion of the three E2 domains I-II from BVDV1a, BVDV1b, and BVDV2, with an N-terminal signal peptide (SP) to facilitate proper protein secretion. (2) The bFc_BVDV_3E2 construct: In this variant, the 3E2 fusion is further modified by incorporating a bovine IgG1 Fc (bFc) domain at the N-terminus, aimed at improving antigen uptake and enhancing immune activation. (3) The bFc_BVDV_3E2_hStab Construct: This design builds upon the second construct by adding STABILON (hStab) element at the C-terminus, thereby enhancing protein conformational stability and extending antigen half-life. (4) The bFc_BVDV_3E2_ARVLP_hStab construct: Integrating the ARVLP element (bovine CD80 TM domain, the guinea pig FcγRII cytoplasmic domain, and the bovine ITCH WWs domain) at the C-terminus of the 3E2 protein and prior to hStab element construction.

The hemagglutinin (HA) _ARVLP construct: WW domain, engineered VLP (containing FcγRII cytoplasmic domain and EABR) or ARVLP (containing FcγRII cytoplasmic domain and WW domain) was fused to the C-terminus of H9N2 or H1N1 HA antigen to compare the expression efficiency and immunogenicity of ARVLP [14,17].

### 2.3. Plasmid Construction, mRNA In Vitro Transcription and Purification

The pUC57 vector (GenScript) was used for plasmid construction and to complete the in vitro mRNA transcription. The T7 promoter element was introduced into the vector to enable the efficient in vitro mRNA transcription. The optimized 5′ and 3′ untranslated region (UTR), a poly(A) tail of approximately 110 nucleotides to enhance mRNA stability and translation efficiency, and the Cap1 structure at the 5′ end was included in mRNA construct. In addition, 6×His tag was incorporated to the C-terminus of ARVLP for antigen detection in vitro. The mRNA in vitro transcription (IVT) was made using MEGAscript^®^ T7 Transcription Kit with CleanCap co-transcriptional capping strategy. Following transcription reaction, mRNA was purified by magnetic beads. Quality control was performed, including mRNA quantification with optical density detection and capillary electrophoresis, to ensure the integrity and purity of prepared mRNA. Finally, mRNA was stored at 4 °C.

### 2.4. Lipid Nanoparticle Encapsulation and Quality Control

We used the microfluidic device to prepare mRNA-LNPs. The lipid components were precisely blended at the following molar ratio: ionizable lipid (SM-102): 50%, distearoylphosphatidylcholine (DSPC): 10%, cholesterol: 38.5%, and methoxy-polyethylene glycol (DMG-PEG2000): 1.5%. The mRNA concentration was prepared at 0.1 mg/mL in 20 mM sodium acetate buffer (pH of 5.5). Controlled nanoparticle assembly was achieved using a microfluidic mixing cartridge, operating at a total flow rate of 6 mL/min and an aqueous-to-organic phase flow ratio of 3:1, with an N/P ratio of 6. Then, the dialysis in 20 mM Tris-HCl buffer (pH of 7.4) and 10% sucrose was performed to ensure stability, homogeneity, and optimal storage. Quality control was completed to ensure the integrity of the mRNA-LNPs. The size distribution of the mRNA-LNPs was measured using dynamic light scattering (DLS), and polydispersity index (PDI) was recorded. Encapsulation efficiency, assessed via the RiboGreen assay (R11490, Thermo Fisher Scientific, Waltham, MA, USA), exceeded 80%, demonstrating effective mRNA encapsulation and protection within the lipid.

### 2.5. Western Blot Analysis

To assess the expression and secretion of the prepared mRNA, HEK293T cells were transiently transfected with mRNA formulated in LNP3 [18]. HEK293T cells were provided by Dr. Yafei Cai. Cells were seeded at a density of 3.75 × 10^6^ per T75 flask; 48 h post-transfection, the supernatants were collected for Western blot (WB) analysis. The collected supernatants were concentrated, and cells were lysed with RIPA. In addition, total protein concentration was determined by BCA assay for further analysis.

Loading buffer (5×) was added to the protein samples. Then, the samples were aspirated and incubated at 100 °C for 5 min. An equal amount of 40 µg per sample was used to conduct sodium dodecyl sulfate-polyacrylamide gel electrophoresis (SDS-PAGE) and protein was transferred to PVDF membranes. Membranes were blocked overnight at 4 °C with 5% skim milk in PBS + 0.05% Tween-20 (PBST) to minimize non-specific binding. For detection, the membranes were incubated with a 1:5000 dilution of a rabbit anti-His tag polyclonal antibody (LF308, Epizyme Biotech, Cambridge, MA, USA) at room temperature (RT) for 1 h. After three PBST washes, the membranes were incubated with an HRP-conjugated goat anti-rabbit IgG secondary antibody (1:5000 dilution in PBST) for 1 h. The chemiluminescence substrate (Immun-Star™ HRP, Bio-Rad, Hercules, CA, USA) was used to detect protein bands, which were visualized with a ChemiDoc™ system (Bio-Rad, USA).

### 2.6. Cryo-Transmission Electron Microscopy Analysis

A portion of the supernatant secreted by cells transfected with H9N2_HA_ARVLP mRNA was purified by size exclusion chromatography (SEC) and subjected to cryo-transmission electron microscopy (cryo-TEM) analysis. Briefly, the 20 mL samples were first concentrated, and the buffer was exchanged using a 100-KDa concentrator with a 15 × 15 × 10 × 10 exchange factor. The resulting ~600 μL concentrate was then evenly mixed with a 200 μL pipette via ten repeated aspirations. Three types of grids were prepared using a glow discharge instrument: Cu300 1.2/1.3 and UltrAu300 1.2/1.3 at 15 mA for 100 s, and Ni-Ti Au 300 1.2/1.3 at 15 mA for 40 s. Next, 3 μL of the solution was applied to each hydrophilized grid and frozen using Vitrobot plunge freezing (4 s blot time, 0 blot force, 30 s hold). Finally, the frozen grids were assembled into cartridges under liquid nitrogen, loaded into the TEM autoloader, and imaged at 92,000× magnification after optical alignment.

### 2.7. Immunization of Mice with mRNA Vaccines

Female BALB/c mice (18–25 g) aged six to eight weeks were procured from Charles River Laboratories (Beijing, China). The mice were acclimated for seven days before the experiment. The mice were assigned randomly to ensure the equitable distribution (n = 5) and a total of 65 mice was used. A total of 40 mice were used for immunization with developed BVDV_3E2 mRNA, bFc_BVDV_3E2 mRNA, bFc_BVDV_3E2_hStab mRNA and bFc_BVDV_3E2_ARVLP_hStab mRNA vaccines. Every single mouse in each group was generally vaccinated intramuscularly with 10 μg vaccine or equal volume of PBS (Control) on day 0 and day 14. Serum samples on days 0 and day 28 were used to analyze neutralizing antibody titers. A total of 15 mice were used for vaccination with H1N1 HA mRNA (vacE), H1N1 HA_ARVLP mRNA (vacF) and PBS. The mice were intramuscularly injected with 10 μg vaccine on day 0 and serum samples on days 0 and 14 were used to analyze neutralizing antibody titers. A total of 10 mice were used to compare immunization route. The mice were injected subcutaneously or intramuscularly with 10 μg bFc_BVDV_3E2_ARVLP_hStab mRNA vaccine on day 0 and day 21. In the above serum samples, on days 0, 28, and 35, samples were used to analyze neutralizing antibody titers, with data from day 0 and/or PBS-injected mice as negative control. In the animal experiments of bFc_BVDV_3E2_ARVLP_hStab mRNA vaccination and subsequent in vivo experiments, there was no separate control group, for the reason that all mice on day 0 could be considered as the control group without immunization, which could reduce the number of mice. After the injection, the mice were observed for potential side effects. Furthermore, serum samples were collected from mice in the same cage on days 0, 28, and 35. These samples were then combined for the purpose of conducting neutralizing antibody titers assays. At the end of the experiment, mice were euthanized by intraperitoneal injection of an overdose of pentobarbital sodium (150 mg/kg) to render them unconscious, in accordance with institutional ethical protocols.

### 2.8. Immunization of Chickens with mRNA Vaccines

A total of 15 of the SPF female laying hens (Suqian 6 strain) aged 90–100 days were selected and divided into 3 groups (n = 5), and every single chicken in each group was immunized by intramuscular injection with PBS (Control), extracellular domain (ECD) of full-length H9N2 HA_ARVLP (vacC), and H9N2 HA mRNA vaccine (vacD) on days 0 and 21, respectively. Serum samples were then collected on days 0, 28, and 35 for determination of hemagglutination inhibition (HI) titers, with data from day 0 and PBS-injected mice as negative control. At the end of the experiment, chickens were euthanized by an intravenous injection of pentobarbital sodium at a dose of 120 mg/kg, in accordance with institutional ethical protocols.

### 2.9. Immunization of Guinea Pigs with mRNA Vaccines

Five SPF female Dunkin-Hartley guinea pigs aged 6–8 weeks, were procured from Charles River and subsequently accommodated within the animal rooms. The animals were provided with a pre-feeding period of seven days. In accordance with the murine experiments, every single guinea pig in each group underwent trivalent mRNA vaccination via intramuscular injection, with a 21-day interval between two injections (n = 5). The administered doses were 20 µg of mRNA per guinea pig. Furthermore, serum samples were collected within the same cage at one-week intervals up to day 56. The antigen-specific antibody titer analysis and neutralizing antibody titer analysis were made for serum samples, with data from day 0 as negative control. At the end of the experiment, guinea pigs were euthanized by an intraperitoneal injection of an overdose of pentobarbital sodium (150 mg/kg) for unconsciousness, in accordance with institutional ethical protocols.

### 2.10. Immunization of Lactating Goats with Optimized BVDV mRNA Vaccines

Nine healthy Laoshan Dairy Goat lactating goats aged 3–4 years were selected to be immunized with trivalent mRNA vaccines in every single lactating goat in each group. We made three immunization strategies (n = 3), which included intramuscular injection, Houhai acupoint injection, and commercial vaccine (Bovine Viral Diarrhea/Mucosal Disease Vaccine, inactivated), with a 21-day interval between two injections. The administered doses were 25 µg of mRNA per goat or 1 mL BVDV Commercial Inactivated Vaccine (Type 1, Strain NM01). Blood samples were drawn from the jugular vein of each goat at 0, 28, 35, 42, 49, and 52 days for neutralizing antibody titer assay, with data from day 0 as the negative control. No lactating goats were anesthetized or unconscious during the experiment. After the experiment ended, the goats continued to be raised.

### 2.11. Hemagglutination Inhibition Titers Analysis

A hemagglutination inhibition (HI) assay was performed using a standard protocol. The serum samples were heat-inactivated at 56 °C for 30 min, treated with receptor-destroying enzyme (C8772, Sigma, St. Louis, MO, USA) at 37 °C overnight, and further inactivated. Two-fold serial dilutions of the serum, starting at a dilution of 1:10, were prepared in a 96-well plate. Then, 4 HA units of the virus were added to each well. After incubating at RT for 30 min, 0.5% chicken red blood cells were added, and hemagglutination patterns were recorded after 30–45 min. The HI titer was defined as the highest dilution of the serum that completely inhibited hemagglutination. Geometric mean titer (GMT) was calculated and log_2_ transformed for statistical analysis.

### 2.12. Neutralizing Antibody Titer Analysis

MDBK cells were commercially purchased from Hefei Wanwu Biotechnology Co., Ltd. (Hefei, China). The cells were cultured in a DMEM medium with 10% fetal bovine serum (FBS) at 37 °C and 5% CO_2_. The serum samples were inactivated at 56 °C for 30 min, then diluted from an initial dilution of 1:8 using a two-fold serial dilution with DMEM containing 2% FBS. An equal volume of the diluted sera was then mixed with 200 TCID50 BVDV-1a (strain NADL), BVDV-1b (strain JS2201), or BVDV-2 (strain C201602) and plated on 96-well culture plates (100 µL/well, four replicates for each dilution). The mixtures were then incubated at 37 °C for 2 h. Thereafter, 1.5 × 10^4^/100 µL of cells were added to each well, and cells were further cultured for 5 days at 37 °C and 5% CO_2_. Virus-infected and uninfected controls without diluted serum were included. The plates were observed daily for the presence of virus cytopathic effects. After 5 days of incubation, the cells were fixed with immune-staining fixing buffer (P0098, Beyotime, Shanghai, China) at RT for 30 min and then washed three times with PBST. The cells were then incubated with blocking buffer (P0102, Beyotime) at 37 °C for 1 h. In this study, the monoclonal antibody against BVDV-1 or BVDV-2 (provided by Dr. Mao Li from the Jiangsu Academy of Agricultural Sciences at a dilution of 1:500) was added to the wells and incubated at 37 °C for 1.5 h. This was followed by an additional 1.5 h incubation period with a FITC-conjugated goat anti-mouse secondary antibody diluted 1:800 (BA1101, Boster, Pleasanton, CA, USA). After washing, positively stained cells were visualized using a Zeiss fluorescence microscope. The neutralization titer for each serum sample was calculated using the Reed–Muench method and expressed as a −log_2_ transformation.

### 2.13. Enzyme-Linked Immunosorbent Assay of bFc-Specific Antibody Titers

An enzyme-linked immunosorbent assay (ELISA) was performed against the bFc domain to determine the IgG titers in the sera of vaccinated guinea pigs. In brief, bovine IgG (36107ES01, Yeasen, Shanghai, China) was coated onto 96-well polystyrene microtiter plates (42592, Corning, Corning, NY, USA) at a concentration of 200 ng/well, as determined by titration, and incubated at 4 °C overnight. After each step, the plates were washed three times with PBST. Blocking was performed at 37 °C for 2 h with PBST and 5% nonfat dry milk. The guinea pig sera were diluted twofold, starting at a dilution of 1:200, and added to the wells in triplicate. The plates were then incubated at 37 °C for 2 h. Rabbit anti-guinea pig IgG/HRP (SE240, Solarbio, Beijing, China) at 1:8000 dilution was used as the conjugate and tetramethylbenzidine (TMB) (PR1200, Solarbio, China) was used as the substrate. After the incubation at 37 °C, absorbance was measured at 450 nm. The ELISA titer is the highest dilution factor at which a serum sample is considered positive.

### 2.14. Protein Structure Prediction and Amino Acids Sequence Alignment Analysis

First, the protein structure of fusion protein of BVDV E2 domains I-II from BVDV1a, BVDV1b and BVDV2 (BVDV_3E2) was predicted by AlphaFold3 (https://alphafoldserver.com) and visualized using PyMOL. Second, the sequences of the WW domains of human, mouse, guinea pig, and chicken ITCH proteins were obtained from the UniProt database. Alignment analysis was then performed using the ESPript3.0 web tool [19].

### 2.15. Statistical Analysis

The GraphPad Prism software (version 10.4.0) was used to analyze the animal data. Parametric tests (e.g., *t*-test, ANOVA) were applied if the assumptions of normal distribution and equal variance were met. Differences between two groups were compared using an unpaired Student’s *t*-test. Differences among multiple groups were analyzed using a two-way ANOVA with a Tukey’s multiple comparisons test. Values of * *p* < 0.05, ** *p* < 0.01, *** *p* < 0.001, and **** *p* < 0.0001 were considered significant.

## 3. Results

### 3.1. Design of a Multivalent mRNA Vaccine Targeting E2 Domains I-II Derived from BVDV Subtypes with Immunogenicity

**Figure 1:**
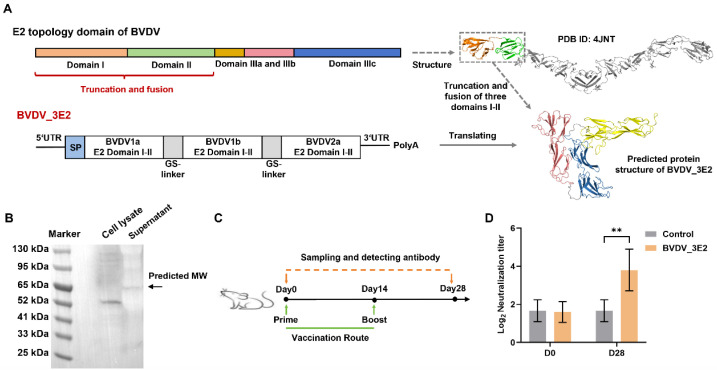
Construction, expression, and immunogenicity analysis of a trivalent BVDV mRNA vaccine containing envelope E2 domains I–II of BVDV1a, BVDV1b, and BVDV2. (**A**) Schematic of the design of the trivalent mRNA vaccine, designated BVDV_3E2. Three of the domains I–II of the E2 protein from BVDV1a, BVDV1b, and BVDV2 were fused and cloned into the mRNA construct, and the predicted protein structure of BVDV_3E2 was obtained by AlphaFold3. (**B**) WB analysis of the BVDV_3E2 protein (predicted MW: 61.7 kDa) in both cell lysate and supernatant following in vitro expression of BVDV_3E2 mRNA. (**C**) Schematic diagram of the experimental design of immunization with BVDV_3E2 mRNA vaccine in BALB/c mice. (**D**) Serum neutralizing antibody titers against BVDV-1a before and after BVDV mRNA vaccination. Data are expressed as means ± standard deviations (SD). Values of ** *p* < 0.01 were considered significant. The original Western blot figures can be found in Supplementary File S1.

Previous studies have demonstrated that the N-terminal immunoglobulin-like domains (domains I-II) of the E2 protein (GenBank accession: 9626650) from BVDV-1a can independently form stable structural scaffolds [11]. To develop an mRNA vaccine targeting BVDV, these domains were truncated to ensure stable IgG-like conformation and immunogenicity that could activate predominant neutralizing capabilities. To address the genetic diversity of BVDV, we synthesized a chimeric construct comprising domains I-II from three prevalent viral subtypes—BVDV1a, BVDV1b, and BVDV2—linked by glycine-serine (GS) linkers to enhance the broad spectrum of the vaccine (Figure 1A). WB analysis revealed a detectable but weak expression of the 3E2 protein (predicted MW: 61.7 kDa) in culture supernatant, while no corresponding intracellular expression was observed (Figure 1B). Subsequently, we immunized mice with the BVDV_3E2 mRNA vaccine, and the analysis of serum neutralizing antibody levels showed that the vaccine activated virus-neutralizing antibodies to some extent (Figure 1C,D). This basic design lays the groundwork for further optimization and evaluation of vaccine efficacy against different strains of BVDV.

### 3.2. Optimization of mRNA Vaccine with IgG1 Fc and STABILON for Enhanced Efficacy Against BVDV

**Figure 2:**
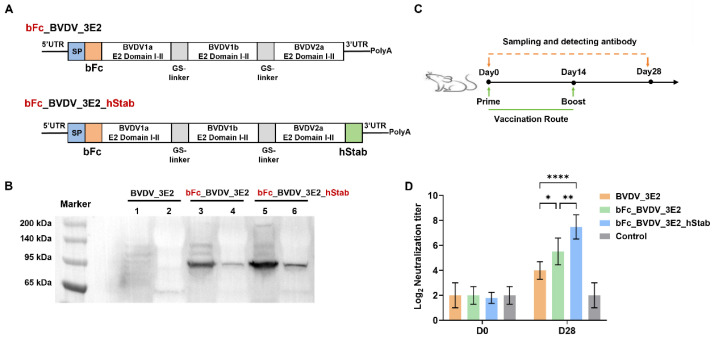
Construction, expression, and immunogenicity analysis of a trivalent BVDV mRNA vaccine encoding E2 domains I–II using bFc and hStab. (**A**) Schematic diagram illustrating the construction of the trivalent mRNA vaccine containing E2 domains I–II derived from three BVDV subtypes with the addition of bFc at the N-terminus or the simultaneous attachment of hStab at the C-terminus. (**B**) Western blot analysis of BVDV_3E2 protein (predicted MW: 61.7 kDa), bFc_BVDV_3E2 protein (predicted MW: 88.6 kDa), and bFc_BVDV_3E2_hStab protein (predicted MW: 90.2 kDa) following mRNA transfection in vitro. Samples in lanes 1, 3, and 5 represent cell lysates, while lanes 2, 4, and 6 contain corresponding culture supernatants. (**C**) Schematic diagram of the experimental design of immunization with mRNA vaccines in BALB/c mice. (**D**) Serum neutralizing antibody titers against BVDV-1a before and after BVDV mRNA vaccination. Data are expressed as means ± SD. Values of * *p* < 0.05, ** *p* < 0.01, and **** *p* < 0.0001 were considered significant. The original Western blot figures can be found in Supplementary File S1.

To enhance protein expression and secretion, we fused the bovine IgG1 Fc domain (bFc) fragment to the N-terminus of the trivalent E2 antigen or simultaneously fused STABILON (hStab) to the C-terminus of the antigen (Figure 2A). The Fc fragment and STABILON have previously been shown to improve antigen trafficking or stability [12,13]. The results of the WB analysis showed that the fusion of bFc fragment significantly increased the intracellular expression levels of 3E2 antigen, and the secretion was also found, which was invaluable for activating the humoral response (Figure 2B). Furthermore, the expression levels in both cells and supernatants were higher with the continued addition of the hStab element (Figure 2B). Analysis of neutralizing antibody titers after immunization of mice showed that bFc and hStab significantly enhanced antibody neutralization potency, demonstrating that these two components can indeed enhance the immune potency of vaccines (Figure 2C,D).

### 3.3. Further Optimization of Trivalent mRNA Vaccine Against BVDV Subtypes with ARVLP

Furthermore, we proposed that the expression of multiple antigens within a single construct may have led to structural instability, which could have hindered higher-level protein expression and secretion. Previously, exosome and VLP were reported to be used as antigen-displaying platforms to improve immunogenicity and stability, in which the WW domains of the Nedd4 family protein could mediate antigen ubiquitination and entry into exosomes; meanwhile, the FcγRII cytoplasmic domain could prevent coated pit localization and endocytosis [14,16]. Therefore, we envisioned the combination of the two structural domains described above to design a type of artificial virus-like particle, i.e., ARVLP, that could enhance the expression and secretion levels by optimizing stability and anchoring the antigen to the membrane of cells and exosomes. The alignment analysis of the sequences of the ITCH (a member of Nedd4 family) WW domains showed high homology among several species (Figure S1A). As shown in Figure S1B, the HA antigen from H9N2 was used as a template to construct three fusion proteins: fusion of HA to the WW domain (vacA), fusion of HA to engineered VLP [14] (vacB), or fusion of HA to ARVLP (vacC) for comparative evaluation. The results showed that the intact ARVLP structure could significantly reduce the expression level of the antigen intracellularly compared to the reported engineered VLP and showed a similarly higher expression level in the supernatant compared to the intracellular (Figure S1C).

**Figure 3:**
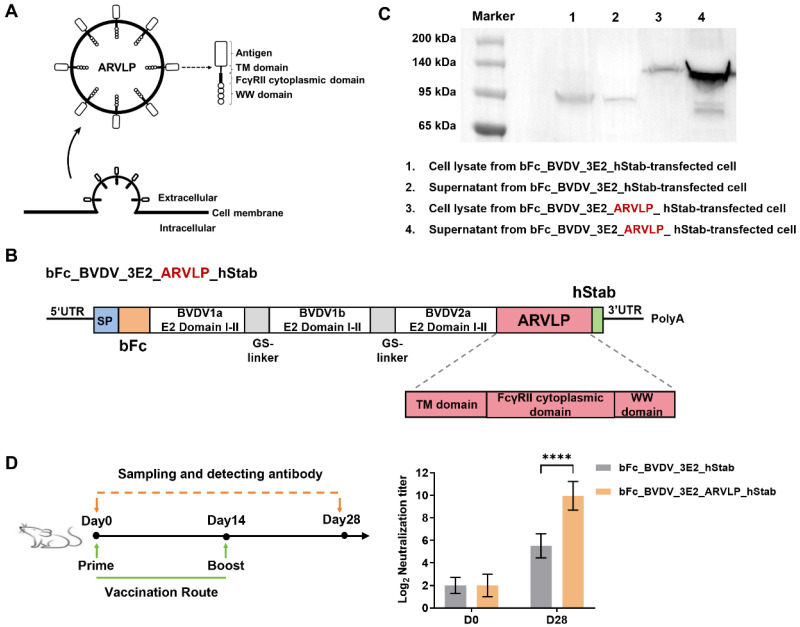
Further optimization of the trivalent mRNA vaccine targeting BVDV with ARVLP. (**A**) Schematic diagram of the formation and composition of ARVLP, in which the TM domain, FcγRII cytoplasmic domain, and WW domain of Nedd4 family protein were fused to the C-terminus of the antigen, and it was secreted by vesicle budding. (**B**) Schematic illustration showing the fusion of ARVLP elements to the bFc_BVDV_3E2_hStab mRNA construct. (**C**) WB analysis of bFc_BVDV_3E2_hStab protein (predicted MW: 90.2 kDa) and bFc_BVDV_3E2_ ARVLP_hStab protein (predicted MW: 124.3 kDa) following mRNA transfection in vitro. (**D**) Schematic diagram of the experimental design of immunization with the indicated mRNA vaccines in BALB/c mice and serum neutralizing antibody titers against BVDV-1a before and after BVDV mRNA vaccination. Data are expressed as means ± SD. Values of **** *p* < 0.0001 were considered significant. The original Western blot figures can be found in Supplementary File S1.

Drawing from the above findings, the combination of the guinea pig FcγRII cytoplasmic domain with WW domain in the C-terminus of antigen containing TM domain showed the best ability to promote antigen secretion. In addition, cryo-TEM analysis revealed that stable VLP structures were found in the supernatant of vacC-transfected cells (Figure S1D). As shown in Figure S2A,B, immunization of chickens with two mRNA vaccines encoding secreted H9N2 HA antigens and analysis of serum HI titers demonstrated that the potency of the serum neutralizing antibodies was significantly increased in the presence of the ARVLP structure. In addition, similar results were obtained in mice immunized with two mRNA vaccines encoding H1N1 HA antigens fused to ARVLP or not (Figure S2C,D). The in vivo immunization experiments demonstrated that ARVLP was indeed effective in significantly enhancing the immunogenicity of the antigen. Therefore, this newly designed ARLVP structure could be used to improve the secretory expression of antigens, and at the same time, it stabilized the structure of displayed antigens and significantly enhance the humoral immune response (Figure 3A).

Finally, we tested the prosecretory effect of ARVLP on bFc_BVDV_3E2_hStab, in which the TM domain of CD80 was fused to ARVLP due to the absence of the TM domain of the antigen (Figure 3B). After in vitro expression, WB analysis showed the higher secretion levels under the condition of adding ARVLP (Figure 3C). In addition, the construct containing ARVLP element further enhanced the immunologic efficacy of the trivalent mRNA vaccine (Figure 3D). Consequently, the bFc_BVDV_3E2_ARVLP_hStab was used as the final vaccine version in the following vaccination experiments.

### 3.4. The Novel Trivalent mRNA Vaccine Elicited Strong Neutralizing Immune Response in Mice

**Figure 4:**
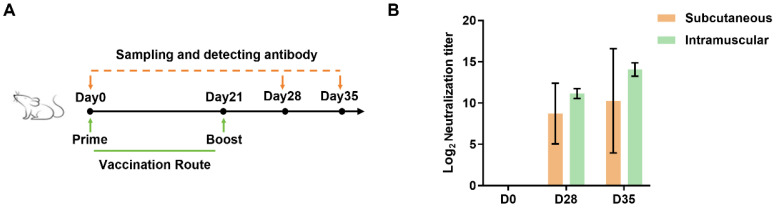
Evaluation of the optimized trivalent mRNA vaccine against BVDV-1a in mice. (**A**) Schematic representation of the vaccination routes and sampling timepoints. (**B**) Serum neutralizing antibody titers against BVDV-1a in mice following subcutaneous or intramuscular administration of the BVDV mRNA vaccine over time. Data are presented as means ± SD.

To evaluate the efficacy of the trivalent mRNA vaccine against BVDV, the bFc_BVDV_3E2_ARVLP_hStab mRNA vaccine was administered to mice by subcutaneous or intramuscular route and serum samples were collected at different time points to assess the development of BVDV-1a neutralizing antibodies (Figure 4A). The humoral immune response was monitored by measuring neutralization titers. The results showed that both routes of vaccination induced robust neutralizing antibody responses against BVDV-1a (Figure 4B). Intramuscular administration resulted in higher peak antibody titers compared to the subcutaneous route, possibly due to differences in antigen distribution and immune cell recruitment. These results demonstrate that the optimized BVDV mRNA vaccine is capable of eliciting a potent neutralizing antibody response and underscore its promise as a viable candidate for broad protection against this economically important viral pathogen.

### 3.5. The Trivalent mRNA Vaccine Elicited Robust and Broad-Spectrum Neutralizing Antibody Responses in Guinea Pigs

**Figure 5:**
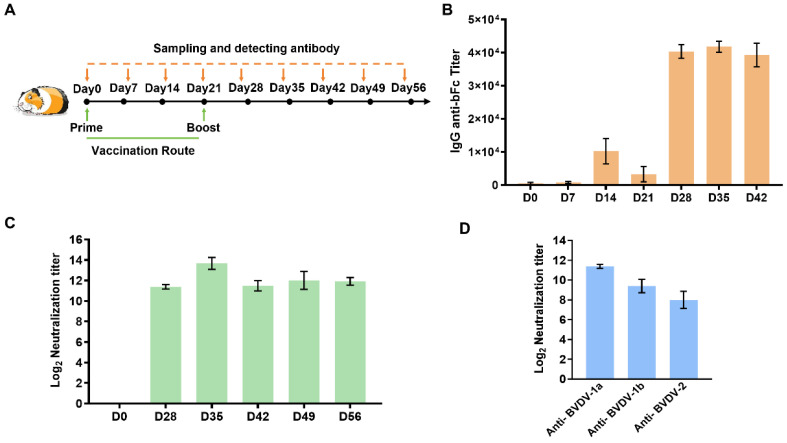
Evaluation of the optimized trivalent mRNA vaccine against BVDV in guinea pigs. (**A**) A schematic diagram of the vaccination and sampling schedule in guinea pigs. Animals received a prime immunization at day 0, followed by a booster dose at day 21. Serum samples were collected at multiple time points (days 0, 7, 14, 21, 28, 35, 42, 49, and 56) for antibody detection. (**B**) ELISA results showing the progression of antigen-specific antibody titers against bFc over time. (**C**) Serum neutralizing antibody titers against BVDV-1a at multiple time points (days 0, 28, 35, 42, 49, and 56). (**D**) Neutralizing antibody titers measured at day 28 against three different BVDV subtypes (BVDV-1a, BVDV-1b, and BVDV-2). Data are presented as means ± SD.

To further evaluate the immune response of the trivalent mRNA vaccine against BVDV in other vertebrates, guinea pigs were immunized with the bFc_BVDV_3E2_ARVLP_hStab mRNA vaccine. As shown in Figure 5A, animals received a primary immunization on day 0, followed by a booster dose administered on day 21. Serial serum samples were collected at regular intervals to quantify antigen-specific IgG levels and assess the durability and magnitude of the humoral response. On days 28, 35, and 42, sera from vaccinated animals showed significantly increased IgG titers to bFc contained in the trivalent mRNA construct (Figure 5B). The decreased antibody titer on day 21 is generally due to an insufficient number of memory B cells after the initial vaccination, resulting in an inability to maintain high antibody levels. We further examined the neutralizing capacity and breadth of the elicited antibodies and observed a marked increase in functional antibodies capable of preventing viral entry and replication on days 0, 28, 35, 42, 49, and 56 (Figure 5C). This indicated a sustained neutralizing antibody response. Next, we delineated the specificity and strength of the neutralizing responses across the three antigenic components encoded by the trivalent vaccine and found that a broad and comparable neutralizing response was elicited against all three BVDV subtypes (Figure 5D).

Taken together, these results indicate that the trivalent mRNA vaccine not only induces strong and durable IgG responses but also confers potent and broad neutralizing activity against different BVDV strains. Such a diverse humoral response provides a compelling rationale for the further development and potential application of this vaccine in the control of BVDV-associated disease.

### 3.6. Broad and Durable Neutralizing Responses Induced by Trivalent mRNA Vaccination in Lactating Goats

**Figure 6:**
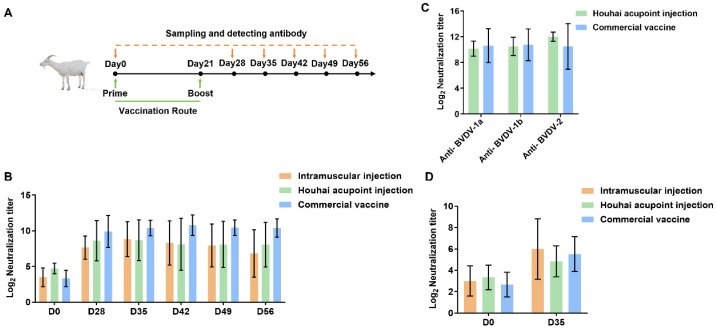
Evaluation of the optimized trivalent mRNA vaccine against BVDV in lactating goats. (**A**) Schematic of the vaccination schedule in lactating goats. Arrows indicate the times of immunization and blood sampling. (**B**) Kinetics of serum neutralizing antibody titers against BVDV-1a after vaccination by intramuscular injection, Houhai acupoint injection, or commercial vaccine administration. (**C**) Cross-neutralizing activity of serum antibodies against different BVDV strains (BVDV-1a, BVDV-1b, and BVDV-2) on day 35 after vaccination. (**D**) Neutralizing antibody titers against BVDV-1a in milk samples from lactating goats on day 0 and day 35 after vaccination. Data are presented as means ± SD.

To evaluate the efficacy of the trivalent mRNA vaccine in large vertebrates, lactating goats were primed on day 0 and boosted on day 21, followed by periodic assessments of neutralizing antibody titers on days 0, 28, 35, 42, 49, and 56 (Figure 6A). All three immunization strategies—intramuscular injection, Houhai acupoint injection, and a commercial vaccine—induced substantial increases in the neutralizing titers relative to baseline, confirming the robust immunogenicity of the vaccine in lactating goats (Figure 6B). By day 35, neutralizing antibody titers obtained by Houhai acupoint injection or intramuscular injection were generally close to those elicited by the commercial standard. By day 56, immunization by the Houhai acupoint injection or commercial vaccine showed sustained high levels of neutralizing antibodies, indicating durable humoral immunity.

Building on these kinetic profiles, vaccine-induced antibodies exhibited broad-spectrum neutralization against multiple BVDV subtypes, including BVDV-1a, BVDV-1b, and BVDV-2, underscoring the ability of the trivalent formulation to provide cross-genotype protection (Figure 6C). Notably, neutralizing antibodies were also detected in the milk of lactating goats, indicating that the induced immunity extended into the lactational environment (Figure 6D). Taken together, these findings highlight the Houhai acupoint injection route as a promising and potentially superior immunization strategy capable of matching or exceeding the performance of a commercially available vaccine while providing broad, durable, and physiologically relevant protection against BVDV.

## 4. Discussion

Currently, inactivated and modified live vaccines are primarily used to prevent BVDV infection in cattle, but this makes it difficult to achieve protection against a wide range of BVDV subtypes, and there are concerns about the safety of attenuated live vaccines. According to previous studies, the BVDV envelope glycoprotein E2 comprises the primary neutralizing epitopes and serves as the pivotal research target for protective antibody responses [9]. Previously, the full-length E2 or fragments were selected as vaccine antigens which yielded suboptimal results, probably due to improper protein folding or exposure of non-neutralizing epitopes [10]. By employing advanced computational structure prediction techniques, we precisely mapped evolutionarily conserved and stable E2 domain I-II across BVDV1a, BVDV1b, and BVDV2 strains. This approach identified immunologically critical domains that remain structurally stable despite viral mutations. These domains adopt a rigid β-sandwich fold that presents key neutralizing epitopes on surface-exposed loops while maintaining proper protein folding [9]. In vitro expression studies confirmed that these rationally selected domains exhibited stability and correct conformational epitopes compared to other E2 fragments. This structure-guided antigen design approach provides a novel foundation for developing broadly protective vaccines.

The development of a multivalent or multi-strain mRNA vaccine represents a significant advance in veterinary vaccinology [20]. For instance, the multi-epitope fusion antigen (MEFA) vaccinology platform had demonstrated remarkable potential in integrating diverse virulence determinants, such as ETEC fimbriae and toxins, into a single immunogen, eliciting broad-spectrum protection against porcine colibacillosis [21,22]. Similarly, the design of multicomponent mRNA vaccines encoding multiple antigens (e.g., MPXV antigens) had shown superior efficacy compared to monovalent formulations, with multivalent combinations conferring robust protection against vaccinia virus (VACV) challenge in preclinical models [23]. These innovations leveraged the inherent flexibility of mRNA technology, which enabled rapid adaptation to circulating strains and the incorporation of conserved epitopes across serotypes, thereby mitigating antigenic variability [24]. However, optimizing multivalent mRNA vaccines requires addressing challenges such as antigen interference, immune dominance hierarchies, and manufacturing complexity. Studies evaluating influenza multivalent vaccines revealed that combining antigens did not always enhance IgG responses or neutralization breadth compared to monovalent counterparts, underscoring the need for rational antigen selection and adjuvant strategies [25,26]. In this study, we truncated the E2 domains I-II of three BVDV subtypes and fused them into trivalent mRNAs, and the results showed that expression of antigen was not affected by the fusion. In addition, bFc, hStab, and ARVLP synergistically increased the level of antigen expression and secretion while enhancing vaccine immunogenicity. The final version of the vaccine expressing ARVLP demonstrated cross-protective immune responses in several animal models, which addresses the genetic diversity of BVDV and potentially provides broader protection against field virus variants.

The effectiveness of mRNA vaccines also depends on achieving stable protein expression and efficient antigen display. To address this challenge, we firstly incorporated strategic modifications including the addition of Fc domain and STABILON [12,13]. Accordingly, Fc could enhance protein secretion through improved folding and trafficking, increase half-life through FcRn-mediated recycling, and potentially enhance immune responses through Fc receptor engagement. These molecular interventions brought by STABILON could enhance protein stability, improve cellular uptake, and potentially extend the immunogenic response duration. Furthermore, the addition of ARVLP containing FcγRII cytoplasmic domain and WW domain significantly enhanced the secretion of fused antigens with display, highlighting the effect on preventing endocytosis and promoting budding and secretion. In addition to the ARVLP used in this study, many engineered exosomes are used to display or encapsulate target antigens to enhance expression and immunogenicity [27]. For example, secretory carrier-associated membrane protein 3 could promote the budding of WW domain-activated extracellular vesicles (WAEVs), in which the proline-proline-alanine-tyrosine motif interacts with the WW domain to recruit fused viral membrane antigens onto WAEVs [17]. Viral membrane proteins displayed on WAEVs induce the production of neutralization antibodies against influenza antigens. The lactadherin VIII-like C1C2 domain is also a widely studied and used engineered exosome element. Komuro et al. used an expression architecture fusing the signal peptide of lactadherin and the C1C2 domain to obtain EVs specific for targeting pancreatic β-cells via the p88 peptide [28]. This shows that the use of engineered VLPs or exosomes as antigen-displaying and targeting vectors has a promising application in vaccine research in other species, including humans.

Meanwhile, this study has some limitations in terms of vaccine and animal experimental designs. For example, whether other conserved BVDV antigens or fragments other than glycoprotein E2 can be used as immunogens to activate broadly neutralizing antibodies needs to be further investigated. In addition, we found that the antibody response activated by Houhai acupoint injection or intramuscular injection was comparable to that of the commercial vaccine. Further studies are necessary to improve the humoral response, including optimization of the delivery vector, dose, and vaccination route. Although the results of the antibody evaluation of the vaccine in goats can serve as a reference for the immunization experiment in cattle, it is still necessary to verify the vaccine’s effectiveness in its primary host. Finally, although neutralizing antibody titer analysis showed that the mRNA vaccine-activated antibodies against glycoprotein E2 effectively inhibited BVDV infection of cells in vitro, the direct role of the vaccine in preventing BVDV infection in animal studies needs to be further investigated.

## 5. Conclusions

In summary, we developed a trivalent mRNA vaccine for the prevention of BVDV infection based on structurally stable tandem E2 domains I–II. The bFc and hStab dramatically enhanced the expression and secretion of fusion antigens. Importantly, the newly designed ARVLP could display the target antigens and effectively enhance the immunogenicity of the antigen. The optimized trivalent vaccine elicited high levels of virus-neutralizing antibodies in several animal models and was cross-neutralizing to all relevant BVDV subtypes. The multivalent engineered vector vaccine developed in this study provides a new idea for the development of viral prophylactic vaccines in livestock and has important clinical research value.

## Data Availability

The data that support the findings of this study are available from the corresponding author upon reasonable request. The GenBank IDs of the constructed gene expression sequences were listed as follows: PV550256 (construct: BVDV_3E2), PV550257 (construct: bFc_BVDV_3E2), PV550258 (construct: bFc_BVDV_3E2_hStab), PV550259 (construct: bFc_BVDV_3E2_ARVLP_hStab), PV550260 (construct: H9N2_HA_WW), PV550261 (construct: H9N2_HA_VLP), PV550262 (construct: H9N2_HA_ARVLP_hStab), PV550263 (construct: H9N2_HA_ECD_Foldon_hStab), PV550264 (construct: H1N1_HA_hStab), PV550265 (construct: H1N1_HA_ARVLP_hStab).

## Supplementary Materials

The following supporting information can be downloaded at: https://www.mdpi.com/article/10.3390/vaccines13070691/s1, Figure S1: comparison of expression efficiency of WW domain or virus-like particle (VLP) fused to hemagglutinin (HA); Figure S2: immunization of animals with HA mRNAs containing or not containing ARVLP; File S1: The original Western blot figures.
