# Supplementary material for: A Novel Trivalent BVDV mRNA Vaccine Displayed by Virus-like Particles Eliciting Potent and Broad-Spectrum Antibody Responses
Source: Vaccines (Basel). 2025 Jun 26;13(7):691. doi: 10.3390/vaccines13070691 (PMC12297935; doi:10.3390/vaccines13070691)
Supplement: Supplementary file 1 [file vaccines-13-00691-s001.zip › vaccines-3686398-File S1. The original Western blot figures.pdf]

Original Images

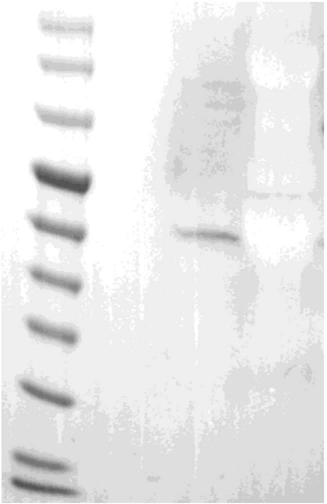

Images in Figure 1B, and lanes from left to right represent samples: (1) marker, (2) loading buffer, (3) lysate, and (4) supernatant from cell transfected with BVDV_3E2 mRNA.

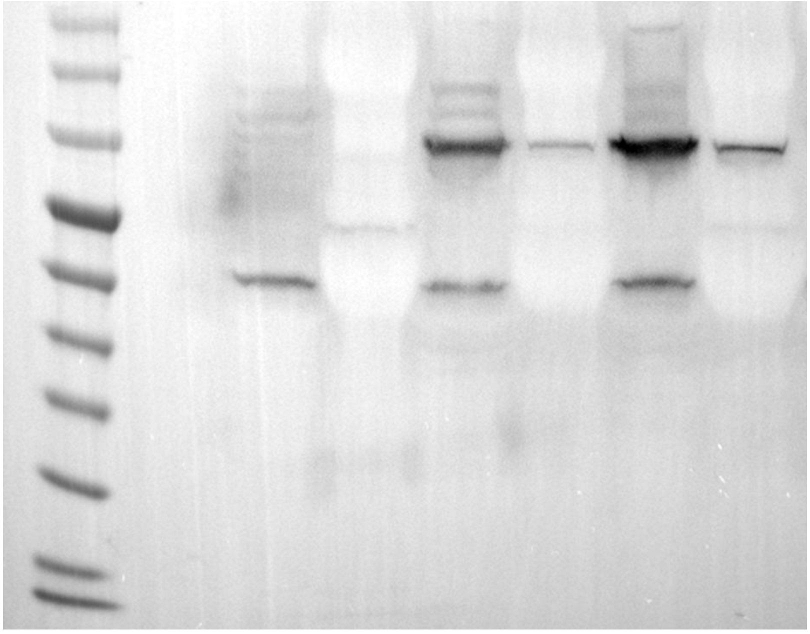

Images in Figure 2B, and lanes from left to right represent samples: (1) marker, (2) loading buffer, (3) lysate, and (4) supernatant from cell transfected with BVDV_3E2 mRNA, (5) lysate, and (6) supernatant from cell transfected with bFc_BVDV_3E2 mRNA, (7) lysate, and (8) supernatant from cell transfected with bFc_BVDV_3E2_hStab mRNA.

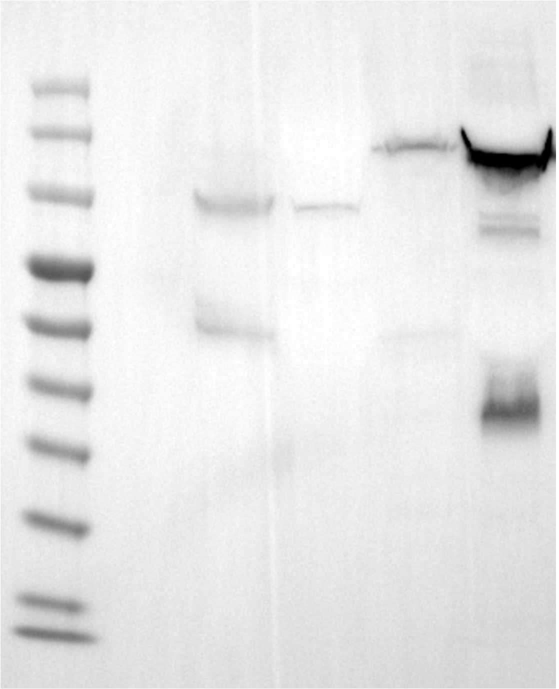

Images in Figure 3C, and lanes from left to right represent samples: (1) marker, (2) loading buffer, (3) lysate, and (4) supernatant from cell transfected with bFc_BVDV_3E2_hStab mRNA, (5) lysate, and (6) supernatant from cell transfected with bFc_BVDV_3E2_ ARVLP_hStab mRNA

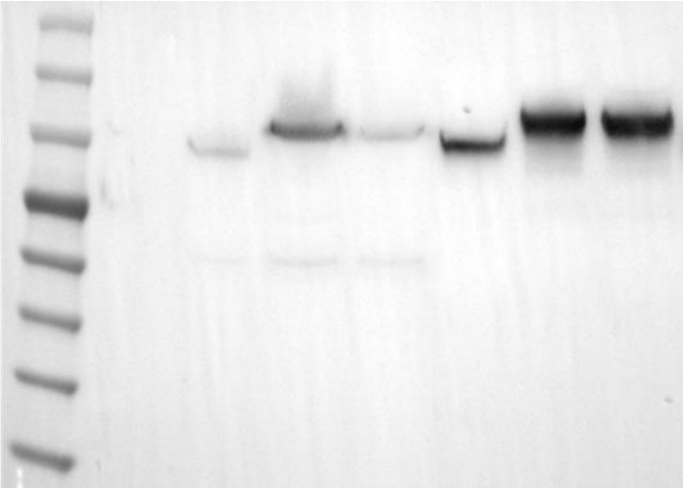

Images in Figure S1C, and lanes from left to right represent samples: (1) marker, (2) loading buffer, lysates from cells transfected with (3) vacA, (4) vacB and (5) vacC, and supernatants from cells transfected with (6) vacA, (7) vacB and (8) vacC.

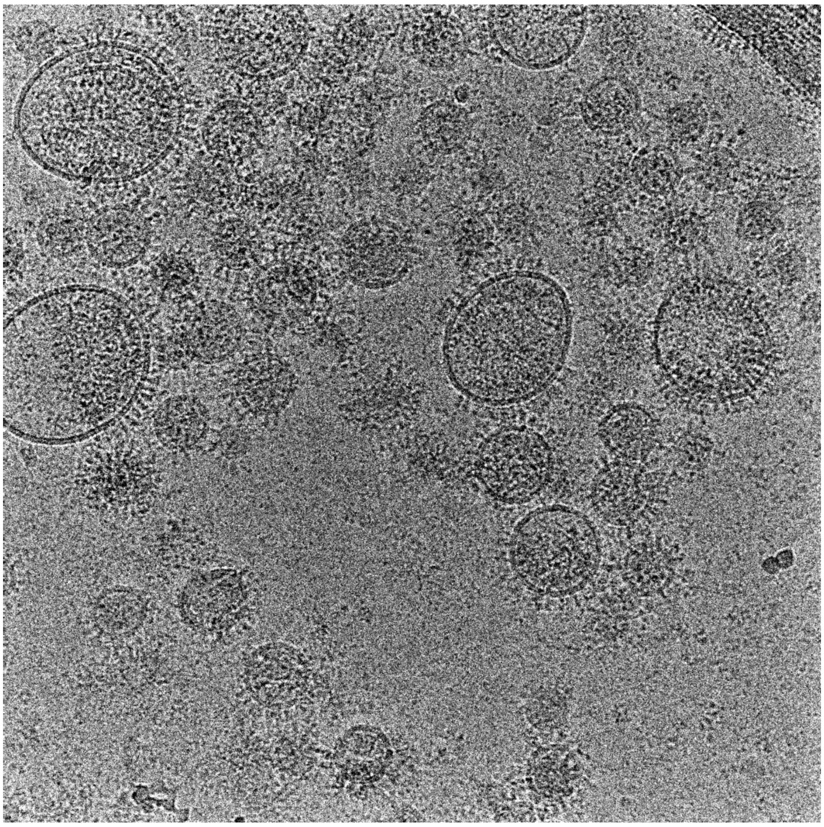

Image in Figure S1D, represent the cryo-transmission electron microscopy (cryo-TEM) capture of ARVLP secreted by vacC.
