# Supplementary material for: A Novel Trivalent BVDV mRNA Vaccine Displayed by Virus-like Particles Eliciting Potent and Broad-Spectrum Antibody Responses
Source: Vaccines (Basel). 2025 Jun 26;13(7):691. doi: 10.3390/vaccines13070691 (PMC12297935; doi:10.3390/vaccines13070691)
Supplement: Supplementary file 1 [file vaccines-13-00691-s001.zip › vaccines-3686398-supplementary.pdf]

Supplementary Information

A novel trivalent BVDV mRNA vaccine displayed by virus-like particles eliciting potent and broad-spectrum antibody responses

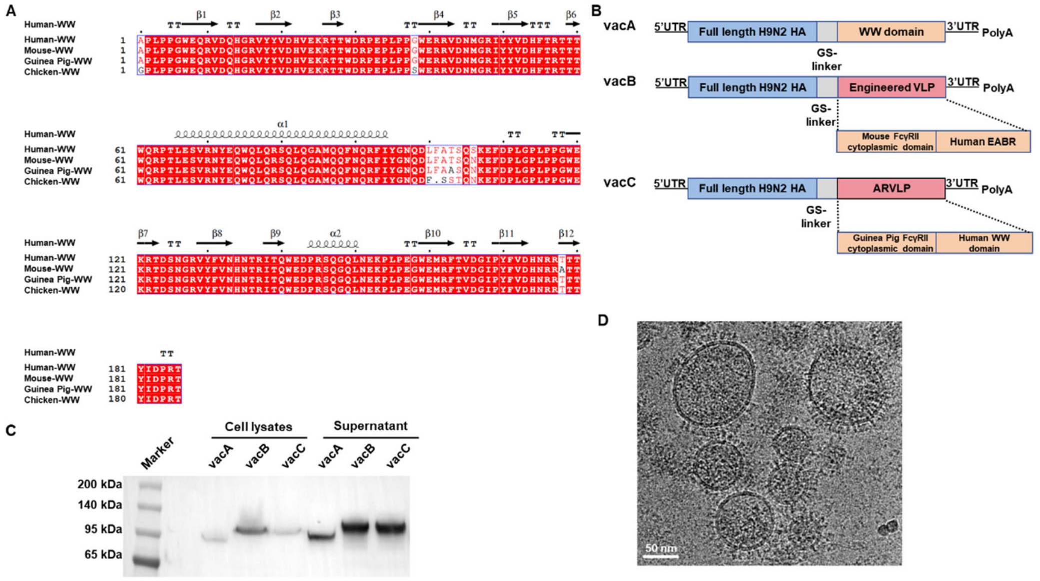

Figure S1. Comparison of expression efficiency of WW domain or virus-like particle (VLP) fused to hemagglutinin (HA). (A) Alignment of protein sequences of different WW domains of human, mouse, guinea pig and chicken ITCH. (B) Construction of indicated mRNA vaccines encoding H9N2 HA fused to WW domain (vacA: H9N2_HA_WW), engineered VLP containing ESCRT and ALIX binding region (EABR) and FcγRII cytoplasmic domain (vacB: H9N2_HA_VLP), or artificial virus-like particle (ARVLP) containing FcγRII cytoplasmic domain and WW domain (vacC: H9N2_HA_ARVLP_hStab). (C) WB analysis of in vitro expression of vacA, vacB and vacC. The predicted molecular weights were 88.6 kDa, 91.8 kDa, and 91.7 kDa, respectively. (D) Cryo-transmission electron microscopy (cryo-TEM) analysis of ARVLP secreted by vacC.

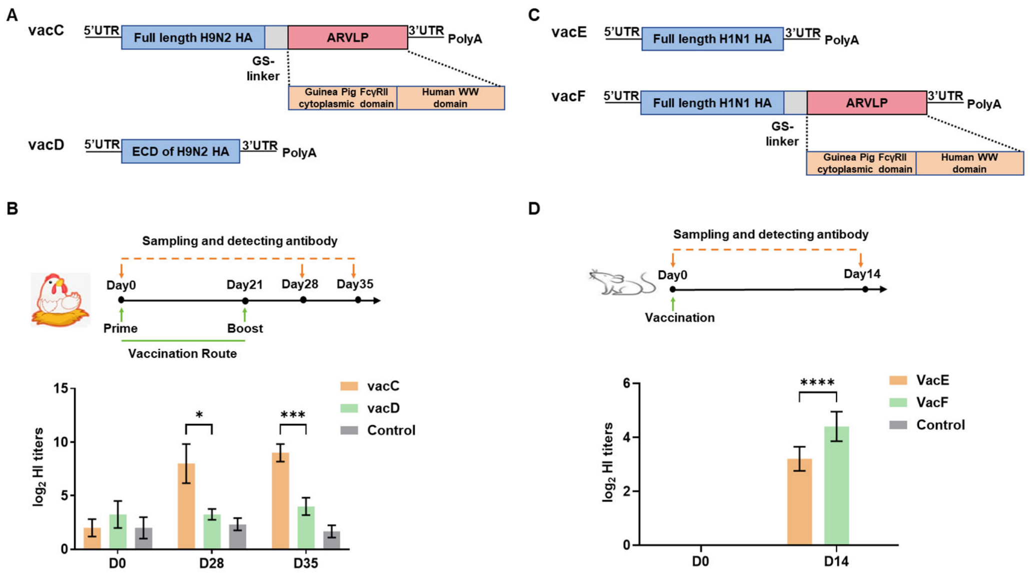

Figure S2. Immunization of animals with different HA mRNAs containing or not containing ARVLP. (A) Schematic diagram of the mRNA construction containing full-length H9N2 HA fused to ARVLP (vacC: H9N2_HA_ARVLP_hStab) or containing the extracellular domain (ECD) of H9N2 HA (vacD: H9N2_HA_ECD_Foldon_hStab). (B) Schematic diagram of the experimental design of immunization with the indicated mRNA vaccines in chicken and analysis of serum hemagglutination inhibition (HI) titers before and after vaccination. (C) Schematic diagram of the mRNA construction containing full-length H1N1 HA (vacE: H1N1_HA_hStab) or containing full-length H1N1 HA fused with ARVLP (vacF: H1N1_HA_ARVLP_hStab). (D) Schematic diagram of the experimental design of immunization with the indicated mRNA vaccines in mice and analysis of serum HI titers on days 0 and 14. Data are expressed as means ± standard deviations (SD). * $p < 0.05$, *** $p < 0.001$, and **** $p < 0.0001$
